# Supplementary material for: Grass-legume mixtures maintain forage biomass under microbial diversity loss via gathering Pseudomonas in root zone soil
Source: mSystems. 2023 Oct 30;8(6):e00755-23. doi: 10.1128/msystems.00755-23 (PMC10734449; doi:10.1128/msystems.00755-23)
Supplement: Supplemental material — Supplemental figures and tables. [file msystems.00755-23-s0001.docx]

*Supplementary information for*

**Grass-legume mixtures maintain forage biomass under microbial diversity loss *via* gathering** **of *Pseudomonas* in root zone soil**

Yu Liu, Tongyao Yang, Yining An, Xiaomeng Li, Hang Gao, Ziheng Peng, Gehong Wei ^*^, Shuo Jiao ^*^

| Target Gene | primer sequence (5’-3’) | amplification procedures |
| --- | --- | --- |
| NifH | nifH-F: AAAGGYGGWCGGYAARTCCACCAC  nifH-R: TTGTTSGCSGCRTACSGCCC | 95°C10min, 40cycles,  95°C 15s, 58°C 30s, 72°C 30s |
| AOA | CrenamoA23F: ATGGTCTGGCTWAGACG  CrenamoA616R:GCCATCCATCTGTATGTCCA | 95°C 5min, 40 cycles,  95°C 30s, 53°C 30s, 72°C 60s |
| AOB | amoAF: GGGGTTTCTACTGGTGGT  amoAR: CCCCTCKGSAAAGCCTTCTTC | 95°C 10 min, 40 cycles,  94°C 30s, 55°C 30 s, 72°C 30s |
| nosZ | nosZ1F: WCSYTGTTCMTCGACAGCCAG  nosZ1R: GTCGCARCTGVKCRTTYTC | 95°C 10min, 40cycles,  94°C 30s, 60°C 60s, 72°C 30s |
| nirS | nirSnF: TACCACCCCGAGCCGCGCGT  nirSnr: GCCGCCGTCRTGVAGGAA | 95°C 10min, 40cycles,  95°C 15s, 63°C 60s, 72°C 30s |
| nirK | NirK583F:TCATGGTGCTGCCGCGKGACGG  NirK909R: GAACTTGCCGGTKGCCCAGAC | 95°C 15min, 40cycles,  95°C 15s, 64°C 60s, 72°C 30s |

**Table S1.** Primers and amplification procedures of functional genes related to the soil nitrogen cycle

**Table S2.** Calculations of Shannon, richness and Inv-Simpson indices for treatments of monoculture and grass-legume mixtures

| Group | Shannon | Richness | Simpson |
| --- | --- | --- | --- |
| M | 5.12±0.40 | 910.00±172.01 | 0.97±0.01 |
| G | 4.90±0.63 | 774.75±299.66 | 0.96±0.02 |
| P | 5.12±0.47 | 914.00±182.71 | 0.97±0.01 |
| GP | 4.99±0.35 | 854.75±128.95 | 0.97±0.01 |
| MG | 5.03±0.71 | 891.50±195.61 | 0.96±0.04 |
| MP | 5.12±0.74 | 1062.75±233.69 | 0.96±0.02 |
| MGP | 5.34±0.51 | 1070.50±147.50 | 0.97±0.02 |

Values indicate the means followed by standard error of the mean. *t*-test showed that the difference between the groups was not significant. **P* ＜ 0.05；***P* ＜ 0.01； ****P* ＜ 0.001.

| Group by comparison | MRPP | |  | ANOSIM |  | PERMANOVA | |
| --- | --- | --- | --- | --- | --- | --- | --- |
|  | Delta | | P | r | P | Pseudo-F | P |
| **M *V.S.* MG** | | 0.6691 | 0.23 | 0.0521 | 0.322 | 1.2212 | 0.231 |
| **M *V.S.* MP** | | 0.631 | 0.215 | 0.1875 | 0.166 | 1.2020 | 0.327 |
| **G *V.S.* MG** | | 0.691 | 0.885 | -0.1458 | 0.951 | 0.8341 | 0.695 |
| **P *V.S.* MP** | | 0.6146 | 0.79 | -0.1146 | 0.823 | 0.8756 | 0.747 |

**Table S3.** Dissimilarity test by MRPP, ANOSIM, and PERMANOVA based on Jaccard dissimilarity between different planting patterns. **P* ＜ 0.05；***P* ＜ 0.01； ****P* ＜ 0.001.


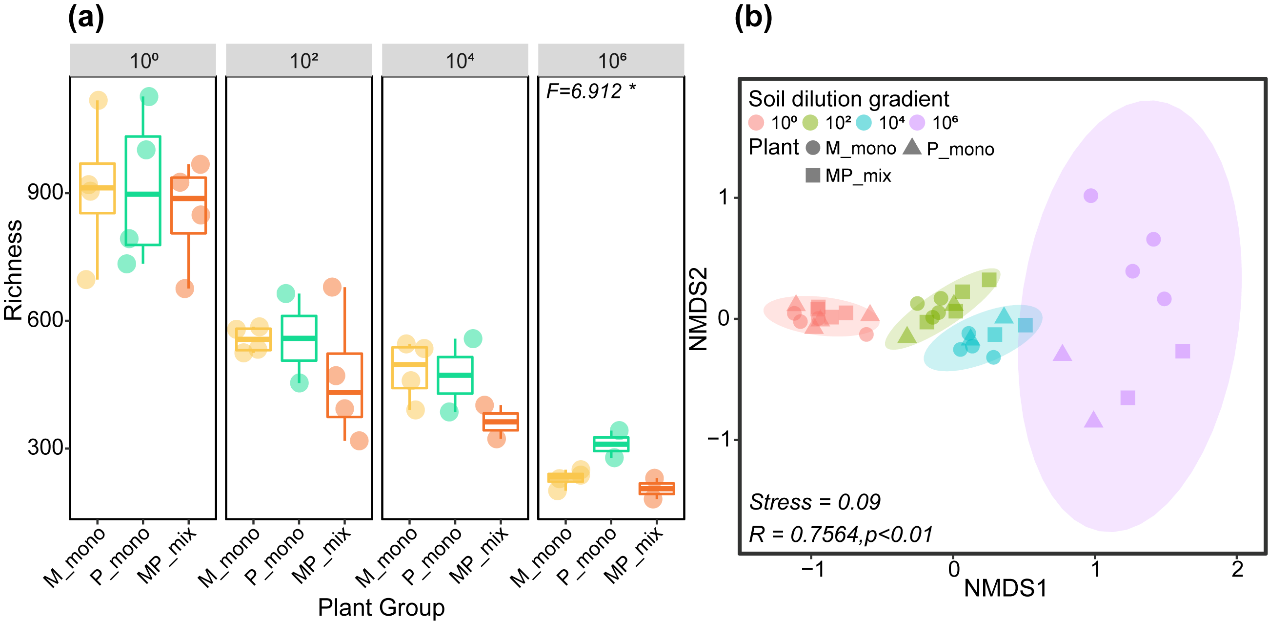
**Fig. S1.** (a) Under the condition of soil microbiome diversity loss, the changes in root zone soil microbial α-diversity were examined across different plant combinations; (b) Under the condition of soil microbiome diversity loss, the changes in root zone soil microbial β-diversity were examined across different plant combinations.


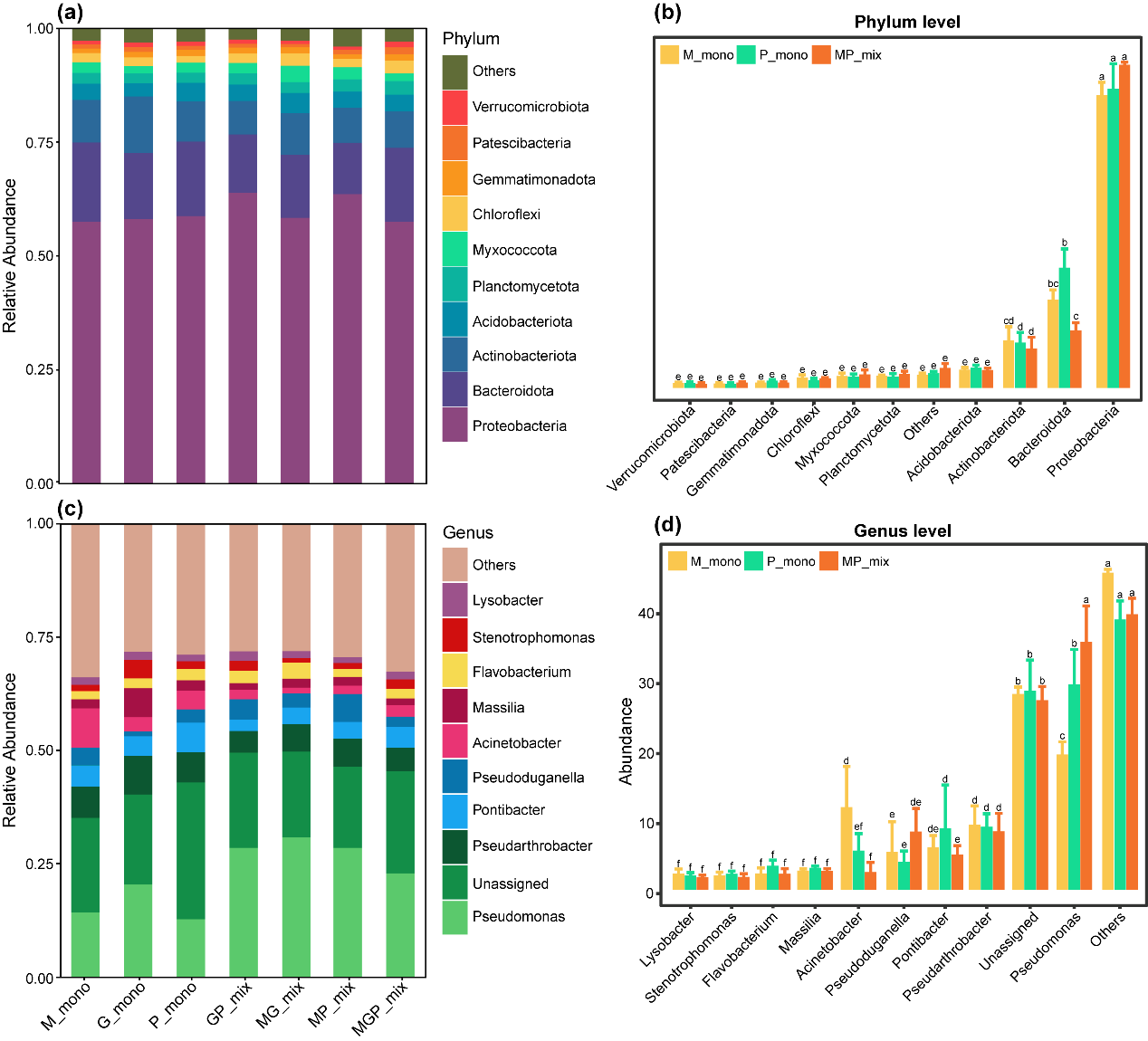
**Fig. S2.** The changes in microbial community composition under different plant combinations. (a) Variation in microbial composition at the phylum level under different plant combinations; (b) Changes in phyla when comparing M_mono, P_mono and MP_mix; (c) Variation in microbial composition at the genus level under different plant combinations; (d) Changes in genera when comparing M_mono, P_mono and MP_mix.


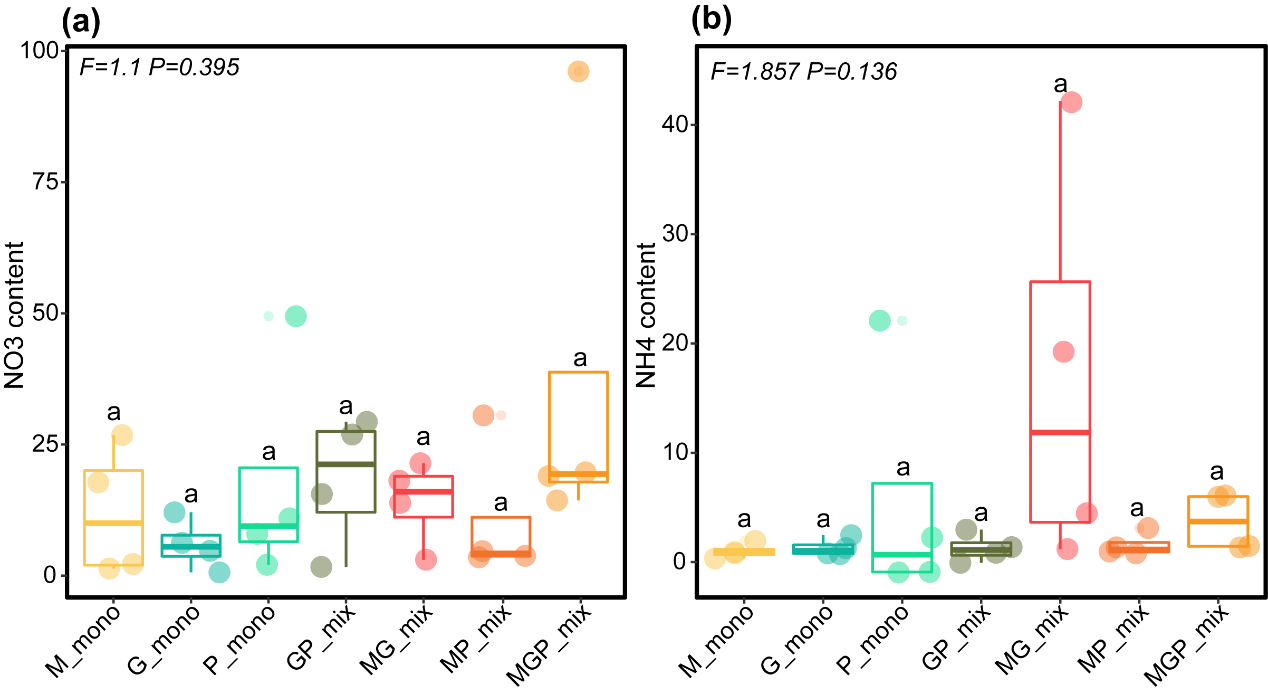
**Fig. S3.** (a) The variations in soil nitrate nitrogen content under different plant combinations; (b) The changes in soil ammonium nitrogen content under different plant combinations.


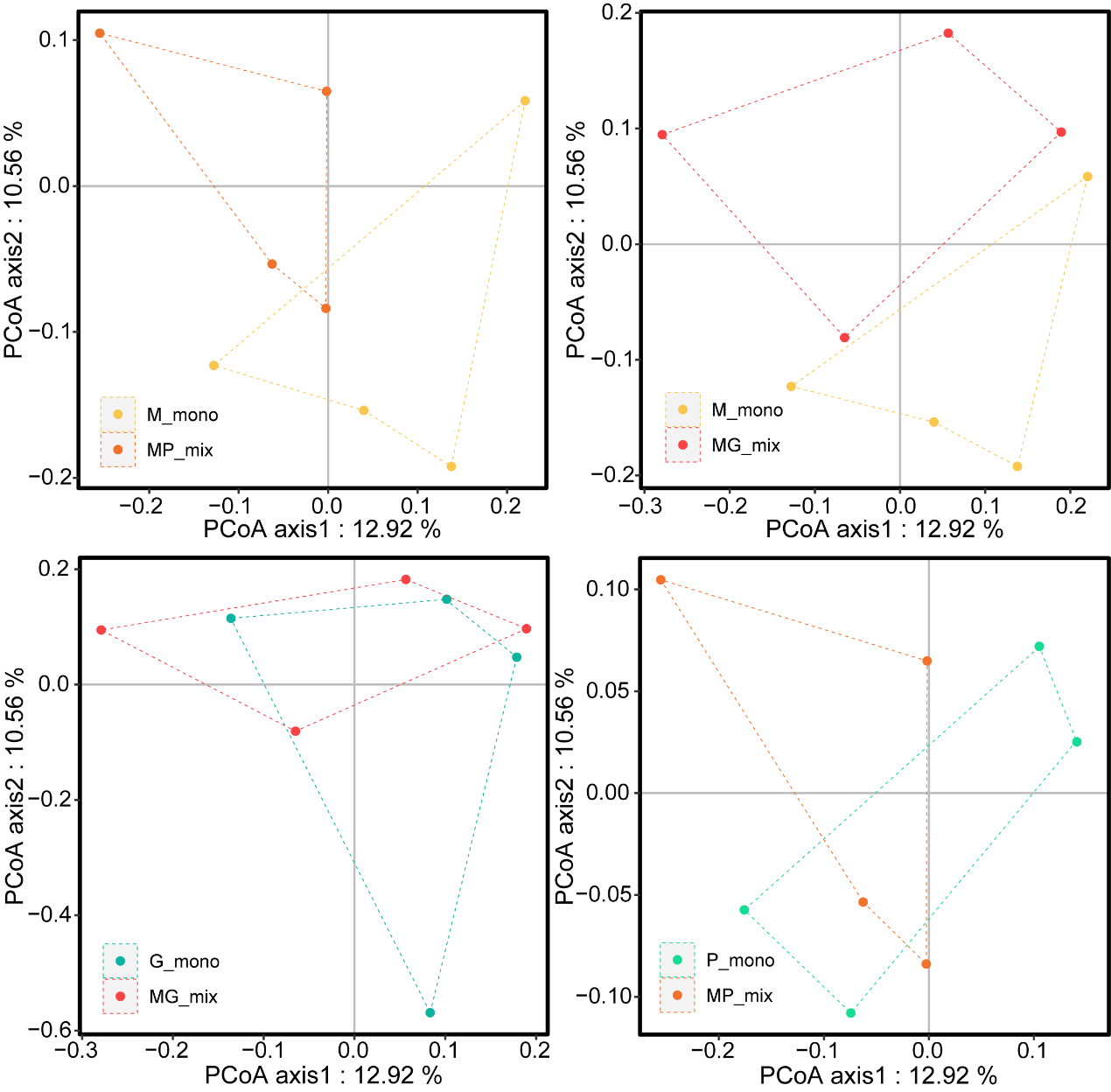
**Fig. S4.** The NMDS plot of bulk root zone microbial communities under monoculture and grass-legume mixtures.


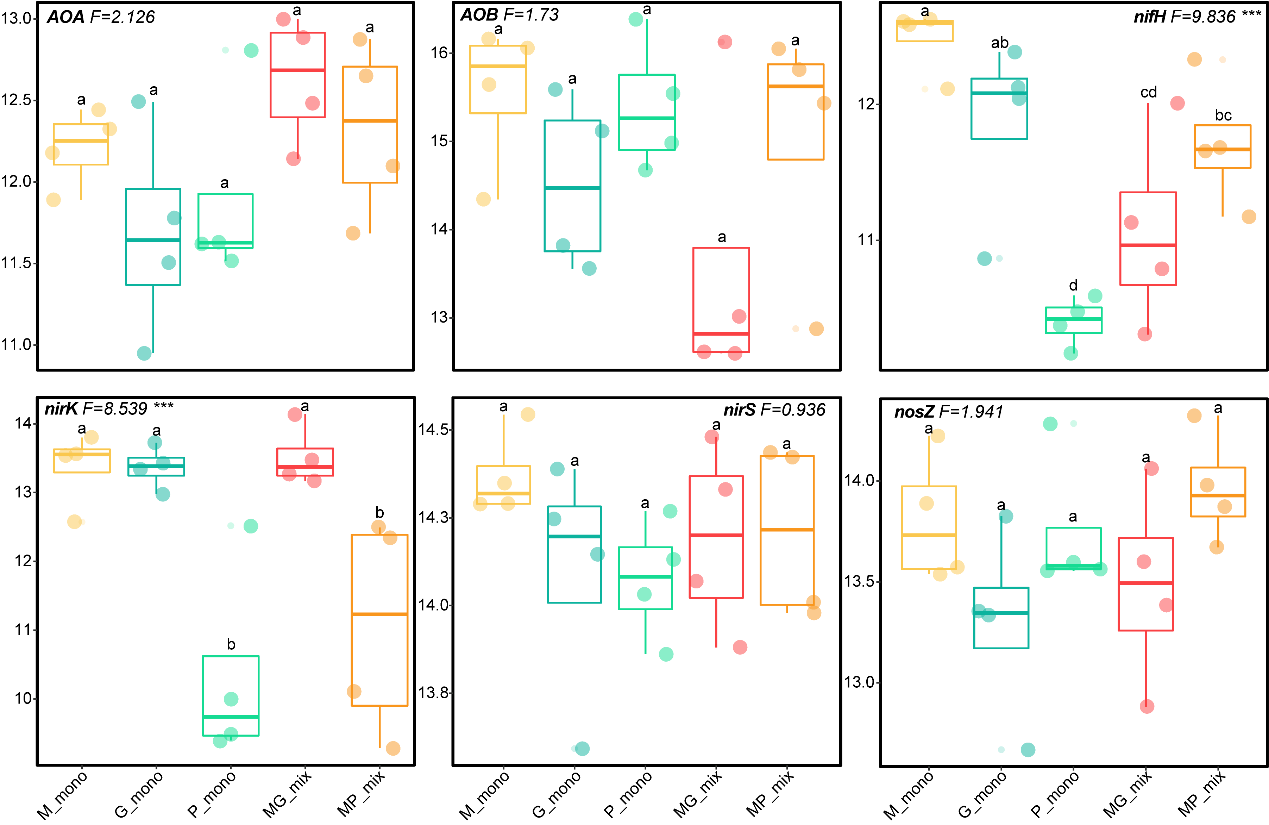
**Fig. S5.** The changes in the abundance of soil nitrogen cycling-related functional genes under different plant combinations.


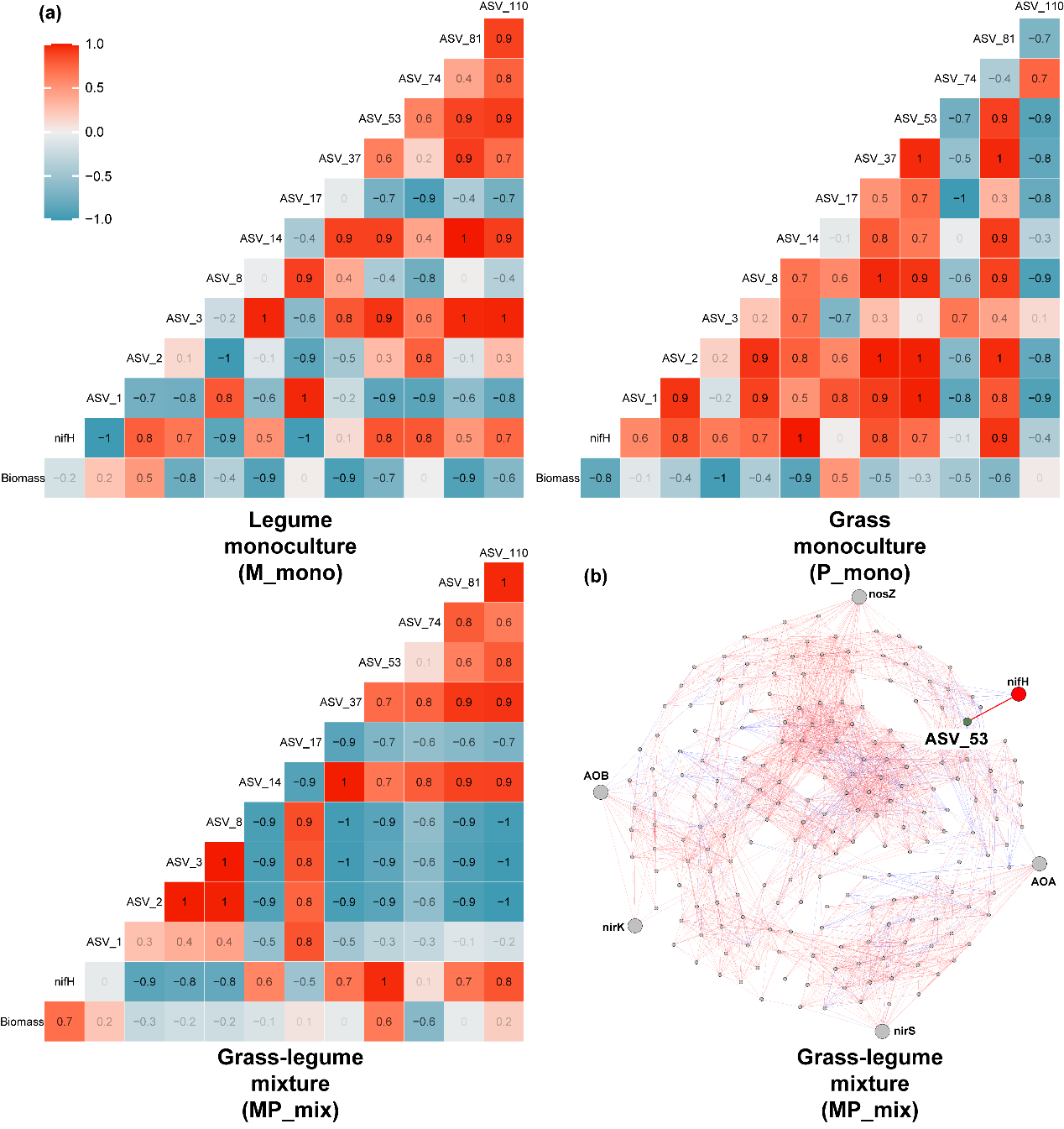
**Fig. S6.** (a) The heatmap demonstrates the correlations between the abundance of ASV53, biomass, and the abundance of nitrogen fixation gene nifH under different plant combinations; (b) Furthermore, by utilizing co-occurrence networks, the relationships between key microbial ASV53 and various nitrogen cycling-related functional genes were explored in the grass-legume mixtures.
